# The Relationship Between Somatization and Depression and Anxiety Levels of Parents with Children Diagnosed with Spina Bifida

**DOI:** 10.1192/j.eurpsy.2023.1227

**Published:** 2023-07-19

**Authors:** P. Ulual, V. Özer, M. Uyar, I. Alatas, G. Özpınar, O. Guclu

**Affiliations:** 1 Psychiatry, Istanbul Basaksehir Cam ve Sakura Sehir Hastanesi; 2 private; 3 neurosurgery, istanbul bilim universitesi; 4 Istanbul Basaksehir Cam ve Sakura Sehir Hastanesi, Istanbul, Türkiye

## Abstract

**Introduction:**

Spina Bifida, a congenital neural tube defect causing multi-system dysfunction. The birth of a disabled child in the family inevitably affects the family members, their lives, feelings, behavior and social life negatively. A lifelong challenge with the disease may give rise to severe pathologies to the parents or caregivers; such as somatization disorder which is characterized by various functional somatic symptoms that can not be explained by organic pathology. For the DSM-V, the diagnosis of complex somatic symptom disorder is proposed to replace the current diagnoses of somatization disorder, undifferentiated somatoform disorder, hypochondriasis and pain disorder. The proposed diagnostic criteria for complex somatic symptom disorder require the presence of somatic symptoms, together with misattributions, excessive concern or preoccupation with symptoms and illness and increased healthcare use.

**Objectives:**

We aimed to find out the relationship between somatization, depression and anxiety levels of parents with children diagnosed with SB.

**Methods:**

Interview form, the Beck Depression Inventory (BDI), Beck Anxiety Inventory (BAI), and the SCL-90-R (Psychological symptoms screening test) were used. 79 individuals were included.

**Results:**

Severely depresssed and anxietic parents show correlating levels of somatizaton. Depression and anxiety scores were above normal range. SCL-90-R Test the ratio of general somatization level compared to other values was found to be 1.72. Parameters above 1 are considered high. This ratio was found to be 100 % in pie charts, indicating all parents had somatization.

**Conclusions:**

SB is not only physical but also a psychological burden to the child as well as the parents. Families often find themselves in despair and feel powerless while giving care to their child with SB. They have a greater tendency for mood and somatization disorder, long term psychiatric follow-up and more frequent evaluations and interventions should be undertaken.

**Disclosure of Interest:**

None Declared

